# COVID-19 and the value of safe transport in the United States

**DOI:** 10.1038/s41598-021-01202-9

**Published:** 2021-11-04

**Authors:** Kenneth B. Medlock, Ted Temzelides, Shih Yu (Elsie) Hung

**Affiliations:** Center for Energy Studies, James A. Baker III Institute for Public Policy, Rice University, Houston, USA

**Keywords:** Energy economics, Environmental social sciences, Energy and society, Socioeconomic scenarios

## Abstract

We investigate the connection between the choice of transportation mode used by commuters and the probability of COVID-19 transmission. This interplay might influence the choice of transportation means for years to come. We present data on commuting, socioeconomic factors, and COVID-19 disease incidence for several US metropolitan areas. The data highlights important connections between population density and mobility, public transportation use, race, and increased likelihood of transmission. We use a transportation model to highlight the effect of uncertainty about transmission on the commuters’ choice of transportation means. Using multiple estimation techniques, we found strong evidence that public transit ridership in several US metro areas has been considerably impacted by COVID-19 and by the policy responses to the pandemic. Concerns about disease transmission had a negative effect on ridership, which is over and above the adverse effect from the observed reduction in employment. The COVID-19 effect is likely to reduce the demand for public transport in favor of lower density alternatives. This change relative to the status quo will have implications for fuel use, congestion, accident frequency, and air quality. More vulnerable communities might be disproportionally affected as a result. We point to the need for additional studies to further quantify these effects and to assist policy in planning for the post-COVID-19 transportation future.

## Introduction

The adverse effects from COVID-19 on the US economy are expected to be present for some time. As of October 14, 2021, the cases in the US has exceeded 44 million (44,715,315) with 720,223 deaths. The costs from hospitalizations, lost economic activity and, especially, loss of life are immense. Importantly, these costs are not borne equally among the US population. For example, those who work remotely can better protect against disease transmission than those who need to commute to work and who spend extended hours in the proximity of others. Furthermore, these effects are correlated with income, race, and socioeconomic status and might give some hints as to why certain segments of the US population have been disproportionally affected by the pandemic. COVID-19 thus exacerbates inequality through disproportionally affecting already vulnerable communities.

Our study concentrates on the interplay between the transportation mode used by commuters in several metropolitan areas in the US and the perceived probability of disease transmission during the commute. This interplay is likely to influence the choice of transportation means for years to come. For example, everything else equal, lower density transportation, associated with lower probability of disease transmission, might be more favored than in the pre-COVID-19 era. This would require additional planning to accommodate the possible increase in demand for some transportation modes and possible decline in others. A shift towards private transport could have large implications for fuel demand, congestion, and pollution control.

There is a small but growing literature on the connections between transportation and the COVID-19 pandemic. Some scholars point to the need for research on how to restore public transportation systems safety and effectiveness in the age of COVID-19^1^. Others give an overview and identify several research questions and policy strategies related to public transportation and COVID-19^2^. It was documented that New York City’s high-ridership subway system highly correlated with COVID-19 transmission during the initial stages of the pandemic in the US^3^. Using linear regressions on county-level data to investigate correlations between COVID-19 deaths and several socio-economic, health, and environmental variables, researchers found that higher levels of commuting via public transportation are correlated with COVID-19 deaths^4^. Using county-level data to investigate racial disparities in COVID-19 mortality, results shows that a significant portion of the disparity can be attributed to the use of public transport^5^. Others used mobile device data to study the relationship between mobility and COVID-19 transmission. The mobile device location data allowed them to compute travel demand for each US county. They found that trips across counties decreased by 35% during the emergency, but recovered rapidly during the partial reopening. They also document a positive relationship between transportation and COVID-19 infections during the pandemic onset and argue that this relationship was stronger in partially reopened regions^6^.

We begin by presenting data on commuting, socioeconomic factors, and COVID-19 disease incidence for US metropolitan areas including the Bay area, Chicago, Miami, Seattle, the DC area, NYC, Portland, San Diego, and Saint Louis. The data highlights important connections between population density and mobility, public transportation use, race, and increased likelihood of transmission. We use a transportation model to highlight the effect of uncertainty about transmission on the commuters’ choice of means of transportation. One implication of our analysis is that individual perceptions of risk, which can in principle depend on age, city, and other population characteristics, or fixed effects, will play an important role in determining commuting choices. Everything else equal, the COVID-19 effect is likely to reduce the demand for public transport in favor of lower density alternatives. Those might include private transport, biking, Uber, or telecommuting. Our estimation results confirm these conclusions are robust across different sample periods, regions, and estimation methods.

We used multiple estimation techniques and found strong evidence that aggregate public transit ridership in each urbanized area (UZA) we studied has been negatively impacted by COVID-19 and by the policy responses to the pandemic, i.e. lock-downs. At the same time, ridership was positively impacted by higher employment levels and a rising price of gasoline. These findings highlight that concerns about disease transmission and COVID-19-related policies have had a negative effect on ridership, which is over and above the adverse effect from the observed reduction in employment. The resulting post-COVID-19 transportation mix will have far-reaching implications for fuel use, congestion and accident frequency, as well as air quality, since local pollutants could increase as a result. Once again, more vulnerable communities would be disproportionally affected. We argue that more studies are needed in order to quantify these effects and to assist policy in planning for the post-COVID-19 future.

## Modeling commuting demand

The possibility of COVID-19 transmission introduces an additional margin when it comes to the choice of means of transport for commuters. Modeling from transportation economics can be used to highlight this choice. We will define the concept of *value-of-avoiding-transmission (VAT)*, which captures the tradeoff between a higher dollar or time cost of transportation and a lower likelihood of disease transmission, perhaps as a result of exposure to a smaller number of travelers, resulting in a lower probability of infection. A central concept in transportation economics is the *value-of-travel-time (VOT)*, which quantifies the well-known tradeoff between saved time and money. More precisely, *VOT* specifies the amount of money that if a commuter had a choice between paying this amount and enjoying a fixed amount of time-savings during his commute, or paying nothing and receiving no time savings, he would be exactly indifferent between the two options. The similar notion of the value of statistical life (VSL) is used in actuarial studies to quantify the tradeoff between reducing the probability of death and a corresponding reduction in income that makes the agent indifferent^7,8^. *VOT* is of central importance in transportation demand modeling, as well as in the cost–benefit analysis of related public policies. For example, it was found that travel time and reliability account for 45% of the average social variable cost of travel in the US^9^.

In the age of COVID-19, there is an additional cost associated with public transportation, namely, an increased probability of exposure to the virus, leading to potential illness and the associated economic costs. When it comes to commuting, these costs can be modeled in a way that is parallel to the costs from potential traffic accidents. Exposure to the virus, just like a traffic accident, occur with some probability in every trip. In addition, just as the probability of an accident increases with congestion, so does the likelihood of infection increase with the number of people using the transportation mode under study. The *VAT* can be used to monetize the desire to reduce the probability of infection by appropriately adjusting the choice of transportation mode.

Transportation studies have explored the relationship between *VOT* and income, wealth, age, time constraints, etc. Travel demand modeling typically finds that travel time is an important explanatory economic variable, even more so than the direct economic cost of travel. The standard model is based on Lave^10^, while more involved theories of *VOT* build on the optimal time allocation framework^11^. People in this framework choose how much labor to supply given a constraint that total time devoted to work, leisure, and commuting equals the total time available to them. Since time can be transferred between work and leisure, any marginal savings in travel time can be used to increase labor income. Intuitively, optimization implies that travel time is valued at the after-tax wage rate. The commuter’s budget constraint can be expressed as

1 $$x + c \le \left( {1 - \tau } \right)w \cdot h$$

while the commuter’s time constraint gives

2 $$l + h + t \le T$$

where *T* is the total available time, *t* is the time spent commuting, *h* corresponds to hours spent at work under after-tax income, $Y = \left( {1 - \tau } \right)w$*,* and $l$ denotes time spent on leisure. Finally, *x* is the expenditure in goods, and *c* is the direct cost of transportation. If the worker uses public transit, *c* would be the public transit fare; if the worker uses private transport, *c* would be the cost of fuel needed for travel; i.e., the price per gallon times miles travelled divided by miles per gallon (or ${{fuel\;price \times miles\;driven} \mathord{\left/ {\vphantom {{fuel\;price \times miles\;driven} { \, fuel\;efficiency}}} \right. \kern-\nulldelimiterspace} { \, fuel\;efficiency}}$). Letting *V* denote the optimal value of the utility function, *u*, the first-order conditions for this problem yield

3 $$VOT = \frac{{{\raise0.7ex\hbox{${\partial V}$} \!\mathord{\left/ {\vphantom {{\partial V} {\partial t}}}\right.\kern-\nulldelimiterspace} \!\lower0.7ex\hbox{${\partial t}$}}}}{{{\raise0.7ex\hbox{${\partial V}$} \!\mathord{\left/ {\vphantom {{\partial V} {\partial h}}}\right.\kern-\nulldelimiterspace} \!\lower0.7ex\hbox{${\partial h}$}}}} = \left( {1 - \tau } \right)w + \frac{{{\raise0.7ex\hbox{${\partial u}$} \!\mathord{\left/ {\vphantom {{\partial u} {\partial h}}}\right.\kern-\nulldelimiterspace} \!\lower0.7ex\hbox{${\partial h}$}} - {\raise0.7ex\hbox{${\partial u}$} \!\mathord{\left/ {\vphantom {{\partial u} {\partial t}}}\right.\kern-\nulldelimiterspace} \!\lower0.7ex\hbox{${\partial t}$}}}}{\lambda }$$

where $\lambda$ is the marginal utility of money. The *VOT* increases with the after-tax wage rate and decreases with the marginal utility of money. This leads to a self-selection where commuters with a higher opportunity cost of time will tend to choose faster, generally more expensive modes of transport.

The recent events related to COVID-19 highlight additional constraints and concerns in connection to public transport. As noted in Figs. 2, 3, 4 and 5 later in "Data visualization" section, there is evidence that the density of public transportation options is highly correlated with an increased probability of transmission of the virus^3–6^. This introduces an additional tradeoff. Increased use of public transport might lead to a higher probability of income loss due to infection and subsequent illness.

Consider a commuter during the COVID-19 era. Every time he uses public transportation, there is a probability, $P\left( n \right)$, of contracting the virus. This probability is increasing in the number of passengers, *n*, since commuter contact—either direct or indirect—with other passengers increases the likelihood of contact with a COVID-19 carrier. Travel time, $t\left( n \right)$, also increases with *n,* since higher capacity utilization implies greater delays. The expected utility for a commuter is given by

4 $$U = P\left( n \right) \cdot u^{V} \left( {Y - F - L} \right) + \left[ {1 - P\left( n \right)} \right] \cdot u^{ - V} \left( {Y - F} \right) - C\left( {t\left( n \right)} \right).$$

In the above expression, *Y* is the commuter’s income, while $u^{V}$ and $u^{ - V}$ stand for the resulting utilities if the commuter is infected and not infected, respectively, during commuting. Infection can lead to medical expenses and lost income from missed work due to mild or severe symptoms, or, in extreme cases, even to death. We denote the resulting expected income loss by *L*, and the commuting fare as *F*. Finally, $C\left( {t\left( n \right)} \right)$ denotes the opportunity costs of commuting travel-time, where ${{\partial C} \mathord{\left/ {\vphantom {{\partial C} {\partial t}}} \right. \kern-\nulldelimiterspace} {\partial t}} > 0$. Thus, increased commuting time adds to commuting costs.

The probability of disease transmission will vary across different means of transport. For example, this probability should be close to zero if one drives their own car to work, especially if not carpooling. The probability will increase when using ride-sharing services or traditional taxis since, although the driver might be the only other person present in the vehicle, disease contagion from previous passengers is still possible. In a bus or train, the probability increases with the number of fellow travelers, *n*.

The model illustrates how infection risk and travel time are linked to commuting density as captured by the number of people, *n*, using this particular means of transport. The marginal change from an increase in the number of commuters can be decomposed into an increase in (a) the implied risk of infection, and (b) the commuting time. The expected marginal utility of income is defined as^8^:

5 $$\lambda = P \cdot \frac{{\partial u^{V} }}{\partial Y} + \left[ {1 - P} \right] \cdot \frac{{\partial u^{ - V} }}{\partial Y}.$$

To avoid an exogenous increase in travel time, commuters would be willing to pay $\frac{1}{\lambda }\frac{\partial C}{{\partial t}}$. This is the standard expression for the *VOT* discussed earlier. The value of an exogenous increase in infection transmission risk is $- \frac{1}{\lambda }\left( {u^{V} - u^{ - V} } \right)$. The value of choosing a transportation mode that implies a marginal reduction in the number of people commuting, thus resulting in a lower probability of infection, is given by

6 $$\frac{1}{\lambda }\left[ {\left( {u^{V} - u^{ - V} } \right) \cdot \frac{\partial P}{{\partial n}} + \frac{\partial C}{{\partial t}} \cdot \frac{\partial t}{{\partial n}}} \right].$$

In this context, we will refer to $\frac{1}{\lambda }\left( {u^{V} - u^{ - V} } \right) \cdot \frac{\partial P}{{\partial n}}$ as the *value-of-avoiding-transmission (VAT)*. Equation (6) captures the combined value of the reduced risk and reduced travel time that would be afforded by a small reduction in the number of people commuting. This could be, for example, the result of using a different (or less crowded) mode for transport. Equation (6) can provide an interpretation for our empirical work related to the marginal rate of substitution between transportation modes associated with different likelihoods of infection.

## Data

Next, we quantify the geospatial and temporal relationship between public transit use and COVID-19 cases. We collected monthly data from January 2010 to July 2020 for the following:

- Unlinked Passenger Trips (*upt*) by National Transit Database (NTD) ID^12^
- Cumulative and new COVID-19 cases by county from USAFacts^13^
- Percent of residents who remain at home by Census block group each month from SafeGraph (which tracks the GPS of mobile devices)^14^
- Total nonfarm employment by county from the Bureau of Labor Statistics^15^
- Retail gasoline prices in selected cities as well as at state and PADD level from EIA^16^.

To begin, we link COVID-19 cases with public transit ridership utilizing available GIS data of public transit routes from the Department of Transport^17^ and county-level data on the incidence of COVID cases. Each public transit agency has a unique NTD ID. Using ArcGIS, we dissolve the routes by NTD ID to obtain the shapes of the public transit network operated by individual agencies. Among the available 174 IDs in the GIS data, we exclude 29 IDs due to incorrect coordinates or attributes, lack of recent data for *upt*, and a lack of a matching metropolitan area in the Bureau of Labor Statistics database. The remaining 145 NTD IDs cover almost 80% of total *upt* nationwide.

In the NTD, each ID corresponds to a specific UZA. In other words, the UZA is the service area of the public transit agency the ID represents. Based on the ID service areas, we aggregate the county level data by NTD ID and match them with *upt* of each NTD ID. We then aggregate the data by UZA by computing the weighted average for each variable based on the population by each NTD ID.

It is important to note that each ID may carry multiple modes of service, such as bus, rail, and ferry boat. In addition, each UZA may have multiple IDs, or transit agencies. For example, the urbanized area of Chicago has 2 IDs—the Chicago Transit Authority (which provides both light rail and bus service) and the Pace-Suburban Bus Division (which provides 4 different modes of transit: demand response, demand response taxi, bus, and vanpool). By aggregating the data by UZA we account for the fact that passengers may switch between multiple available public transit services in the same urbanized area. There are 94 unique UZAs (see Table 1) among the 145 NTD IDs that presented with complete data. Moreover, 18 out of the 94 UZAs host more than one NTD ID.

**Table 1:** UZAs by city, state designation(s).

1	Seattle, WA	33	Nashville-Davidson, TN	65	Austin, TX
2	Spokane, WA	34	Raleigh, NC	66	Lincoln, NE
3	Yakima, WA	35	Lexington-Fayette, KY	67	Kansas City, MO-KS
4	Eugene, OR	36	Sarasota-Bradenton, FL	68	St. Louis, MO-IL
5	Portland, OR-WA	37	Cape Coral, FL	69	Des Moines, IA
6	Biose City, ID	38	Miami, FL	70	Salt Lake City-West Valley City, UT
7	Anchorage, AK	39	Tallahassee, FL	71	Sioux Falls, SD
8	Longview, WA-OR	40	Jacksonville, FL	72	Denver-Aurora, CO
9	Kennewick-Pasco, WA	41	Birmingham, AL	73	Missoula, MT
10	Olympia-Lacey, WA	42	Montgomery, AL	74	Logan, UT
11	Bremerton, WA	43	Durham, NC	75	Urban Honolulu, HI
12	Bellingham, WA	44	Charleston-North Charleston, SC	76	San Francisco-Oakland, CA
13	Medford, OR	45	Columbia, SC	77	Bakersfield, CA
14	Wenatchee, WA	46	Appleton, WI	78	Santa Cruz, CA
15	Boston, MA-NH-RI	47	Milwaukee, WI	79	Los Angeles-Long Beach-Anaheim, CA
16	New Bedford, MA	48	Oshkosh, WI	80	Stockton, CA
17	Pittsfield, MA	49	Akron, OH	81	San Jose, CA
18	Hartford, CT	50	Cleveland, OH	82	San Diego, CA
19	Leominster-Fitchburg, MA	51	Columbus, OH	83	Riverside-San Bernardino, CA
20	Barnstable Town, MA	52	Lansing, MI	84	Tucson, AZ
21	Buffalo, NY	53	Ann Arbor, MI	85	Oxnard, CA
22	New York-Newark, NY-NJ-CT	54	Peoria, IL	86	Yuba City, CA
23	Syracuse, NY	55	Davenport, IA-IL	87	Santa Maria, CA
24	Ithaca, NY	56	Champaign, IL	88	Santa Rosa, CA
25	Lynchburg, VA	57	Chicago, IL-IN	89	Fairfield, CA
26	Pittsburgh, PA	58	Eau Clarie, WI	90	Davis, CA
27	Washington, DC-VA-MD	59	Elkhart, IN-MI	91	Concord, CA
28	Baltimore, MD	60	Dallas-Fort Worth-Arlington, TX	92	Santa Clarita, CA
29	Philadelphia, PA-NJ-DE-MD	61	Houston, TX	93	Merced, CA
30	Chattanooga, TN-GA	62	Oklahoma City, OK	94	Flagstaff, AZ
31	Knoxville, TN	63	Tulsa, OK		
32	Memphis, TN-MS-AR	64	Albuquerque, NM		

The percentage of people who remain at home each month, $PctHm_{t}$, is estimated by SafeGraph using mobile phone tracking data. This data is only available beginning in January 2019, but no massive, exogenous, pandemic-induced shocks occurred between January 2010 and the beginning of 2020. So, we calculate a year-over-year difference in the percentage of people who remain at home for each month, $PctHm_{t} - PctHm_{t - 12}$, and multiply the resulting series by a dummy variable, $D_{COVID19,t}$, that takes a value of 1 for all months in which new cases of COVID-19 are reported in each UZA, and a value of 0 otherwise. This is represented as

$$DevPctHm_{t} = \left( {PctHm_{t} - PctHm_{t - 12} } \right)D_{COVID19,t} .$$

We then use the calculated value $DevPctHm_{i,t}$ for each UZA in our estimation analysis.

## Data visualization

**Figure 1:**
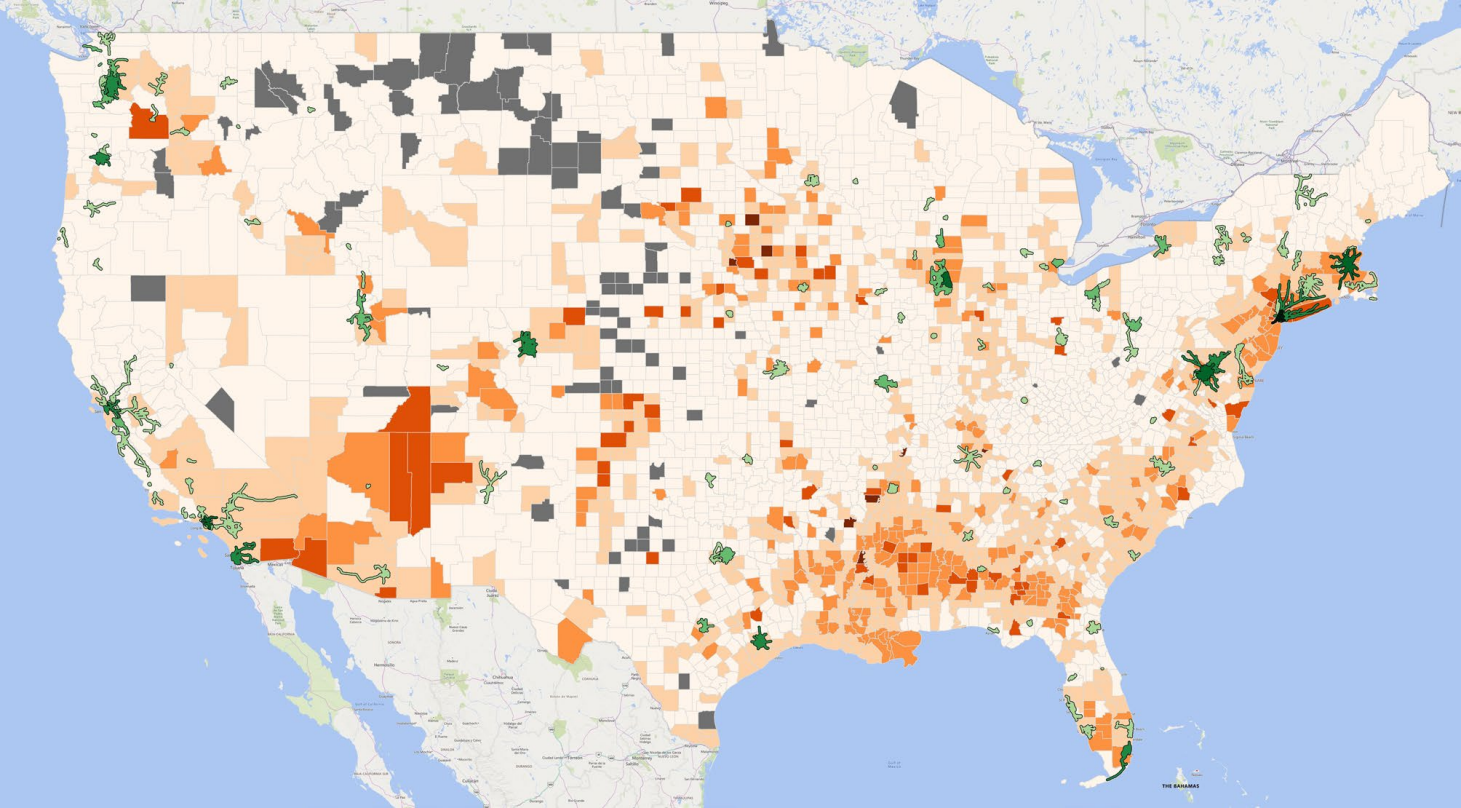
COVID-19 cases per 1000 population by county and the public transit systems in the US, 6/30/2020. The heatmap in orange incidates cumulative COVID-19 cases per 1000 population as of June 30, 2020 by county with public transit agency routes (in shades of green) using the data from the National Transit Database. Both data sets are categorized into 5 classes using Jenks natural breaks. Darker orange suggests higher COVID-19 cases, and darker green indicates higher use of the public transit system. Counties that are grayed-out had zero reported COVID-19 cases on this date. The map was created by the authors with QGIS 3.12 (https://qgis.org/en/site/forusers/download.html).

For a national perspective of COVID-19 cases and to connect to the public transit routes and their use, in Fig. 1 we overlay a heat map of cumulative COVID-19 cases per 1,000 population as of June 30, 2020 by county (in shades of orange) with public transit agency routes (in shades of green) using the data from the National Transit Database. Both data sets are categorized into 5 classes using Jenks natural breaks. Darker orange suggests higher COVID-19 cases, and darker green indicates higher use of the public transit system. Counties that are grayed-out had zero reported COVID-19 cases on this date.

**Figure 2:**
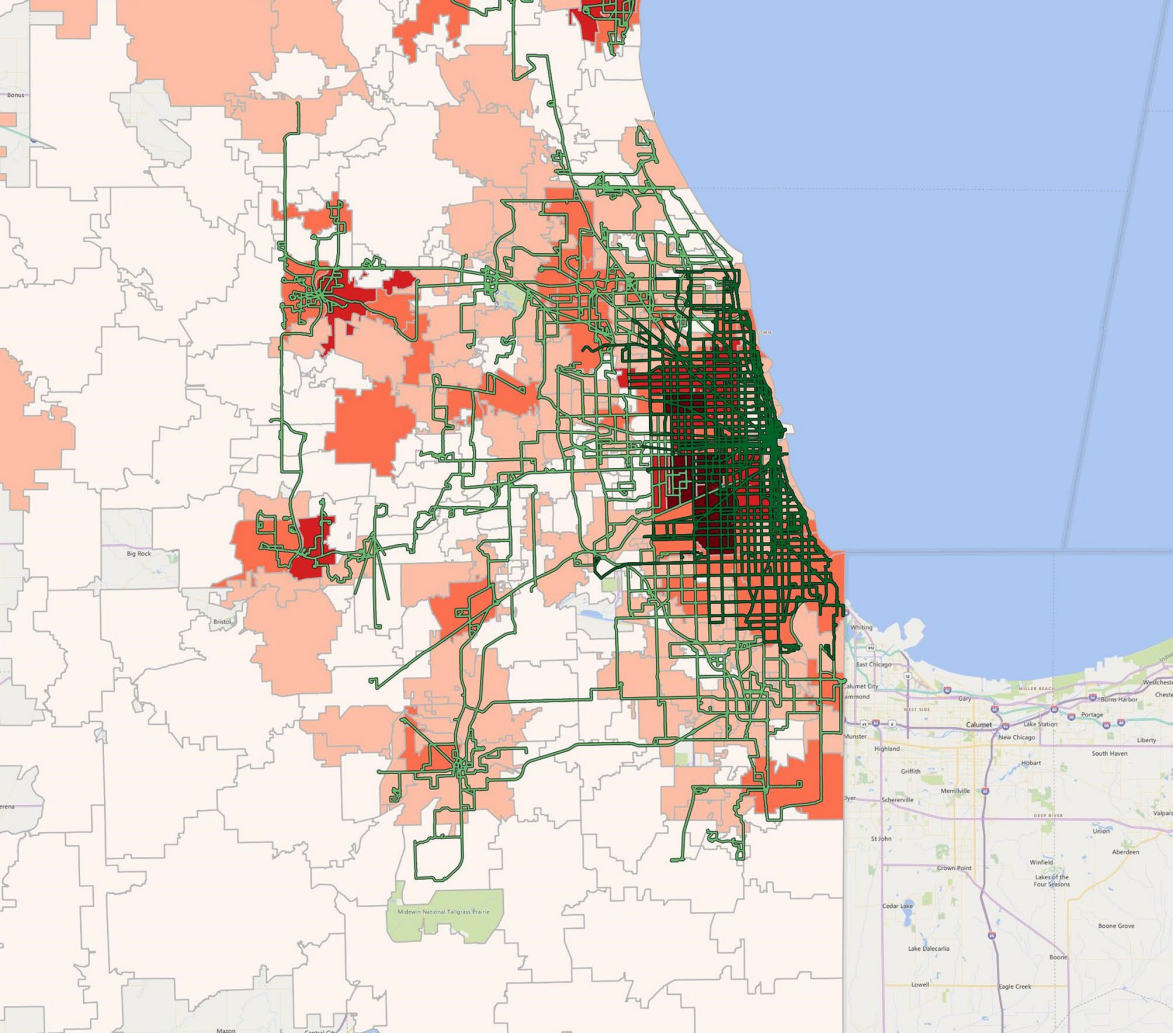
COVID-19 cases and the public transit system in Chicago, 6/30/20. The heat map suggests COVID-19 cases by zip code (in shades of orange) overlaid with public transit routes. The number of cases is the lowest in pale orange and highest in dark red The map was created by the authors with QGIS 3.12 (https://qgis.org/en/site/forusers/download.html).

**Figure 3:**
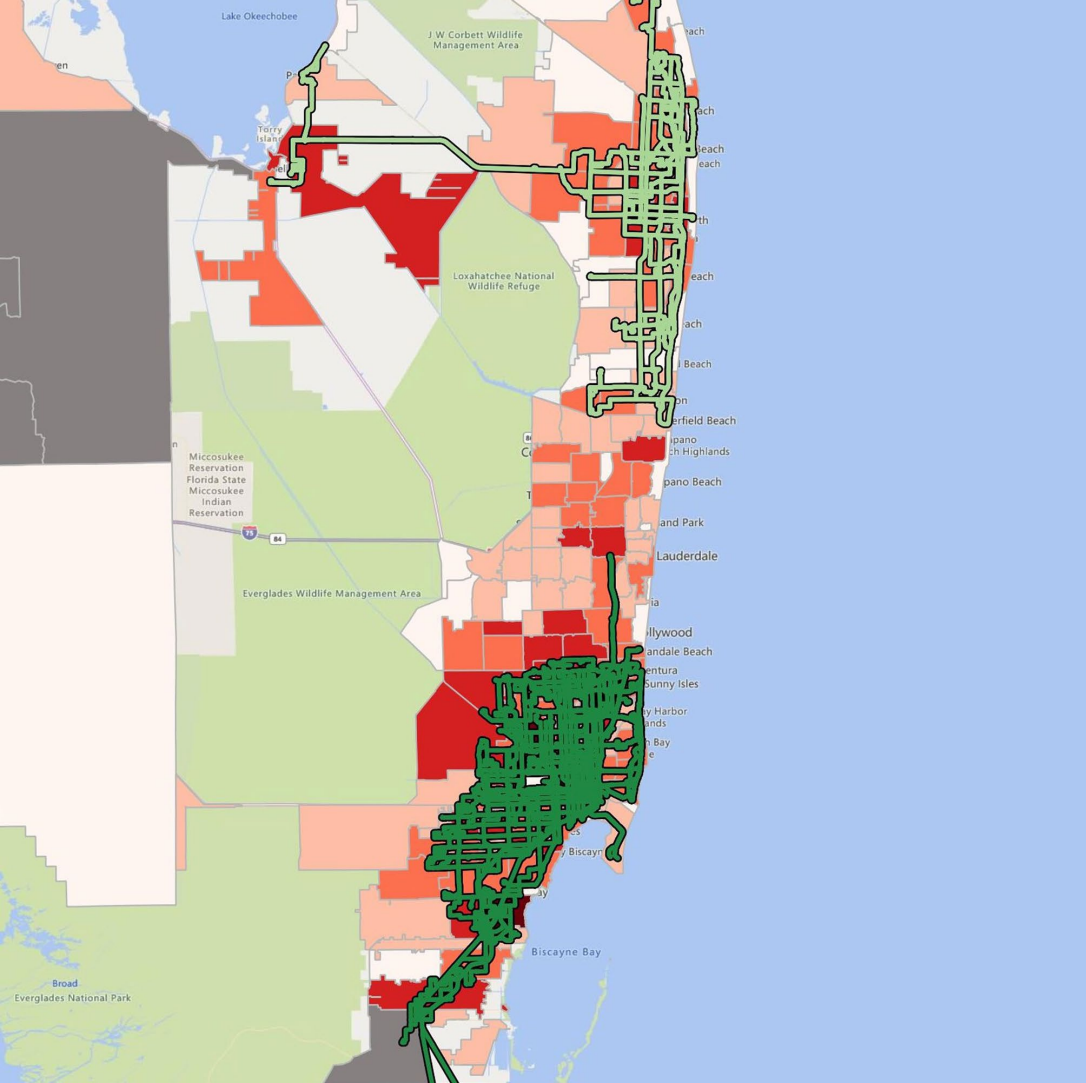
COVID-19 cases and the public transit system in Miami-Jupiter, 6/30/20. The heat map suggests COVID-19 cases by zip code (in shades of orange) overlaid with public transit routes. The number of cases is the lowest in pale orange and highest in dark red. The map was created by the authors with QGIS 3.12 (https://qgis.org/en/site/forusers/download.html).

**Figure 4:**
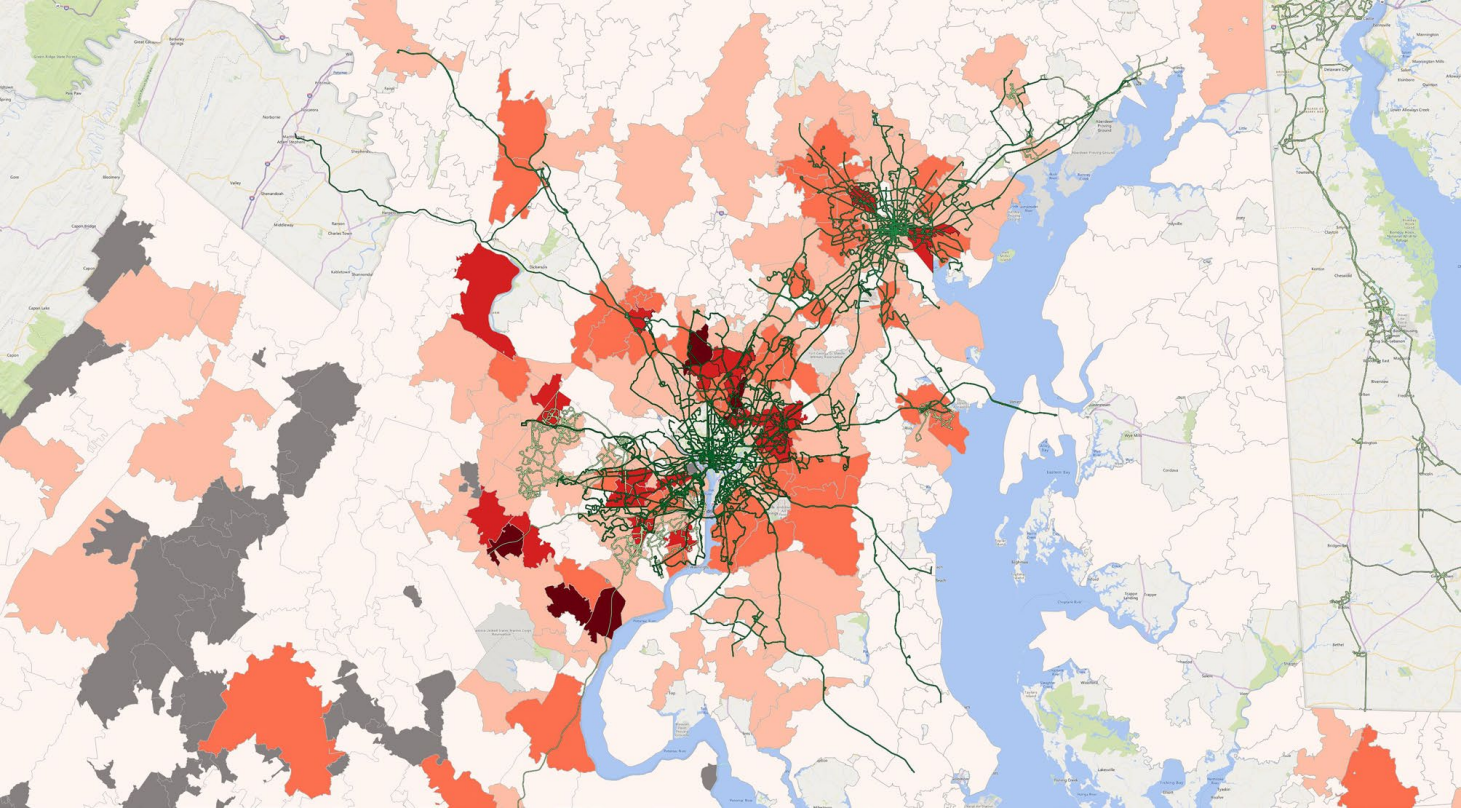
COVID-19 cases and the public transit system in Washington DC area, 6/30/20. The heat map suggests COVID-19 cases by zip code (in shades of orange) overlaid with public transit routes. The number of cases is the lowest in pale orange and highest in dark red. The map was created by the authors with QGIS 3.12 (https://qgis.org/en/site/forusers/download.html).

**Figure 5:**
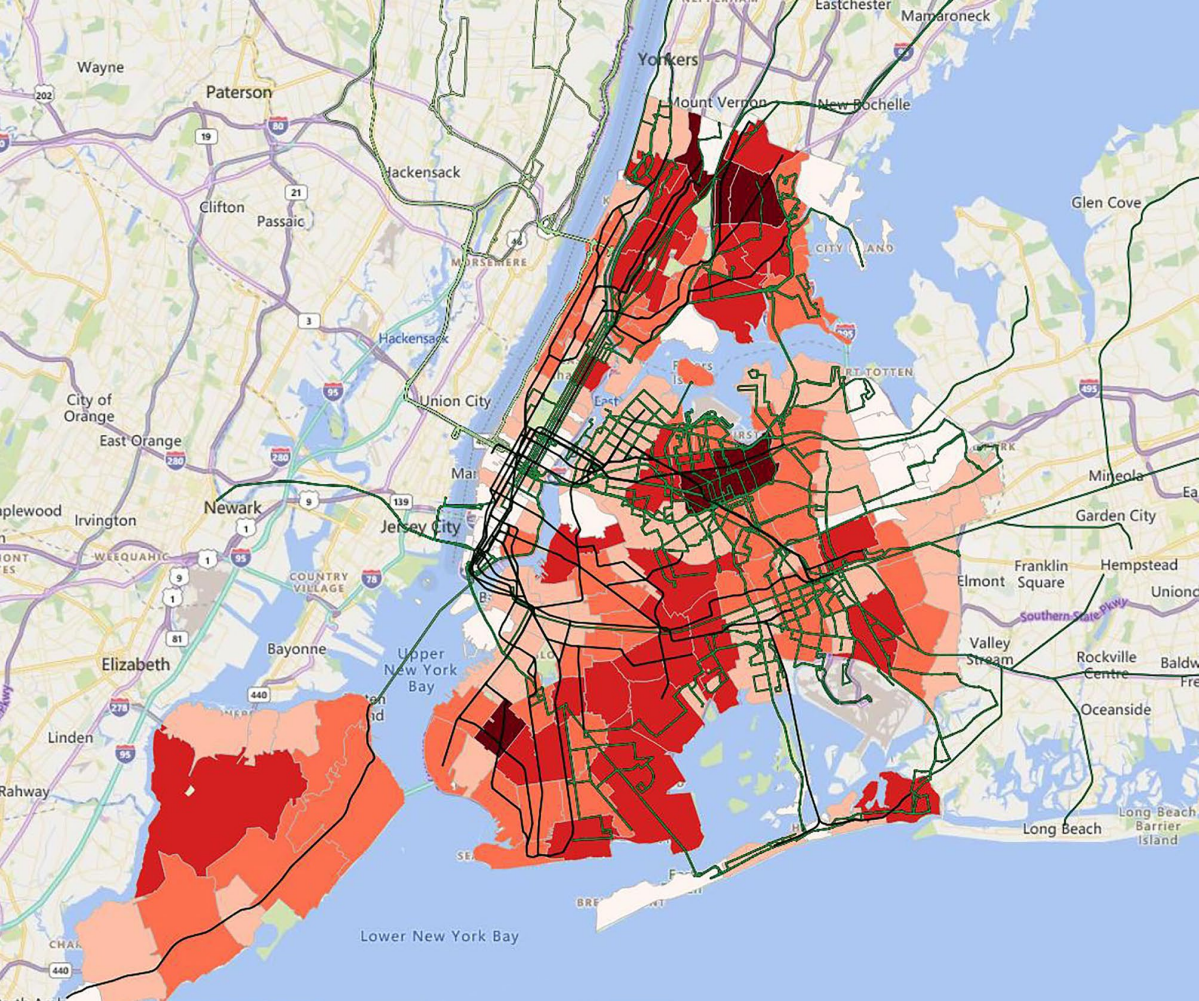
COVID-19 cases and the public transit system in New York-Newark, 6/30/20. The heat map suggests COVID-19 cases by zip code (in shades of orange) overlaid with public transit routes. The number of cases is the lowest in pale orange and highest in dark red. The map was created by the authors with QGIS 3.12 (https://qgis.org/en/site/forusers/download.html).

Next, we use higher granularity data to focus on major metropolitan areas. In Figs. 2, 3, 4 and 5, we indicate the Chicago, the Miami-Jupiter, the Washington DC, and the New York-Newark UZAs (additional UZAs are presented in the Appendix). We produced the maps using the aforementioned method—a heat map of COVID-19 cases by zip code (in shades of orange) overlaid with public transit routes. The number of cases is the lowest in pale orange and highest in dark red. Although this is no proof of a causal relationship, zip codes with higher cases highly correlate with the areas covered by local public transit systems, especially where the public transit network is the densest.

**Figure 6:**
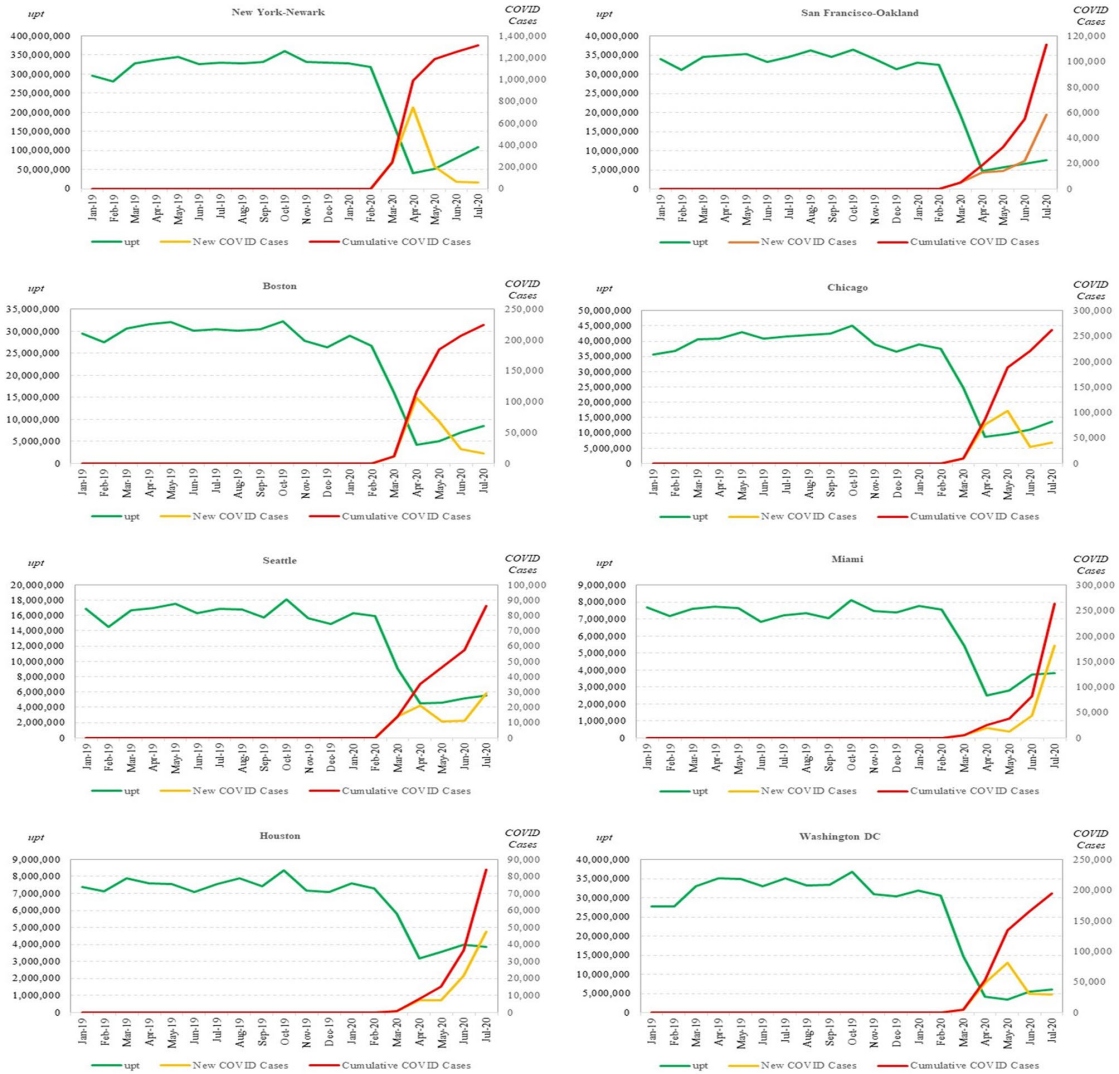
UPT and COVID cases in 8 Select UZAs (Jan 2019–Jul 2020). The left axis indicates the number of UPT (unlinked passenger trips) in green, while the right axis indicates both the number of new COVID cases in yellow and the number of cumulative COVID cases in red.

In Fig. 6, we plot available monthly data for *upt*, cumulative COVID cases, and new COVID cases from Jan 2019 to Jul 2020 for 8 select UZAs: New York-Newark, San Francisco-Oakland, Boston, Chicago, Seattle, Miami, Houston, and Washington DC. We see that *upt* in each UZA were dramatically impacted by COVID outbreaks, and the situation was still evolving as of July 2020.

## Estimation and results

We estimate the following expression:

7 $$\begin{aligned} \ln upt_{i,t} & = \alpha_{0} + \alpha_{1} \ln upt_{i,t - 1} + \alpha_{2} DevPctHm_{i,t} + \alpha_{3} newCOVID_{i,t} \\ & \quad + \alpha_{4} \ln N_{i,t} + \alpha_{5} \ln p_{gas,i,t} + \sum\limits_{j = 6}^{16} {\alpha_{j} D_{j} } + \beta_{i} + \varepsilon_{t} \\ \end{aligned}$$

where $\ln upt_{i,t}$ denotes the natural logarithm of unconnected passenger trips at time *t* in region *i*, $\ln N_{i,t}$ denotes the natural logarithm of employment at time *t* in region *i*, $DevPctHm_{i,t}$ denotes the deviation from historical norms of the fraction of people remaining at home at time *t* in region *i*, $newCOVID_{i,t}$ denotes the number of new COVID cases at time *t* in region *i*, and $\ln p_{gas,i,t}$ denotes the natural logarithm of the price of gasoline at time *t* in region *i*. In addition, the variables $D_{j}$ are monthly dummy variables taking a value of 1 in month *j* and 0 in all other months, while $\beta_{i}$ is a region-specific constant (or fixed effect) to be estimated along with all $\alpha_{j}$. Finally, $\varepsilon_{t}$ is a normally distributed error term.

The variables $DevPctHm_{i,t}$ and $newCOVID_{i,t}$ capture the impacts that COVID-19, both through direct commuter avoidance of public transit and policy responses that mandated business and office closures and/or limited in-person activity. Referencing the discussion in "Modeling commuting demand" section, these are also key indicators of the value of avoiding transmission (VAT). Employment is included to capture the impact of labor income and the aggregate demand for transportation services for transit to and from work. The price of gasoline is included to account for the price of private transportation services, which serves as a proxy for the cost of transportation services that compete with public transit options. We also include monthly dummy variables to account for any seasonality in public transit use.

We estimate Eq. (7) using both the Arellano–Bond (AB) and Instrumental Variables (IV) estimation techniques for a dynamic panel with fixed effects. The Hausman specification test indicates the difference in coefficients between the fixed effects and random effects estimators is systematic. We instrument for both the lagged endogenous variable and employment to control for the any endogeneity of the two variables and the other included regressors. We chose a dynamic specification to account for habit persistence in commuter behavior. The fixed effects allow for persistent regional differences that reflect, among other things, socioeconomic variables such as income, demographics, poverty levels, commuting patterns, etc.

Both estimation methods are consistent with each other in terms of marginal impacts of the included variables, and each confirms a strong correlation between COVID-19 incidence and use of public transport. As instruments we use a single lag of the right-hand side variables. All estimations were done in STATA. The panel is balanced, with each of the 94 UZAs including 125 observations each, accounting for the use of lagged variables, spanning the period January 2010 to July 2020. We present the results of the AB estimator here, but a table with full set of the results using the IV estimator, along with the AB estimator is in the Appendix. AB estimation of Eq. (7) yields

8 $$\begin{aligned} \ln upt_{i,t} & = \mathop {4.2303}\limits_{(0.1973)} + \mathop {0.6173}\limits_{(0.0080)} \ln upt_{i,t - 1} - \mathop {1.5767}\limits_{(0.0329)} DevPctHm_{i,t} - \mathop {0.0013}\limits_{(0.0002)} newCOVID_{i,t} \\ & \quad + \mathop {0.1140}\limits_{(0.0293)} \ln N_{i,t} + \mathop {0.1276}\limits_{(0.0096)} \ln p_{gas,i,t} + \mathop {0.1230}\limits_{(0.0066)} D_{Jan} + \mathop {0.0937}\limits_{(0.0064)} D_{Feb} + \mathop {0.1668}\limits_{(0.0065)} D_{Mar} \\ & \quad + \mathop {0.0390}\limits_{(0.0065)} D_{Apr} + \mathop {0.0544}\limits_{(0.0065)} D_{May} - \mathop {0.0085}\limits_{(0.0065)} D_{Jun} + \mathop {0.0177}\limits_{(0.0066)} D_{Jul} + \mathop {0.1423}\limits_{(0.0067)} D_{Aug} \\ & \quad + \mathop {0.1308}\limits_{(0.0066)} D_{Sep} + \mathop {0.1848}\limits_{(0.0066)} D_{Oct} + \mathop {0.0109}\limits_{(0.0066)} D_{Nov} + \hat{\beta }_{i} . \\ \end{aligned}$$

All included variables are statistically significant at the 1% level, except the monthly dummy variables for June and November, the latter of which is significant at the 10% level. Note that December is dropped due to the inclusion of the constant, so the month of December is the point of comparison for each month. The pattern of the monthly effects is the same across the two estimation methods, indicating a seasonal effect where public transit ridership is higher in March, August, September and October. Note, these are nationwide averages as the fixed effects will capture any regional differences across UZAs.

The implications of Eq. (8) are fairly straightforward. Namely, *upt*, which is our aggregate measure of public transit ridership in each UZA, has been negatively impacted by COVID-19 and related policies. It is positively impacted by higher employment levels and a rising price of gasoline. Note, this is precisely what one would expect from the theoretical framework presented in "Modeling commuting demand" section above. This is due to VAT considerations that make high-density public transport less attractive to commuters. It also highlights the impacts that COVID-19 and related policies have had on ridership, which is over and above the reduction in employment that has occurred.

**Figure 7:**
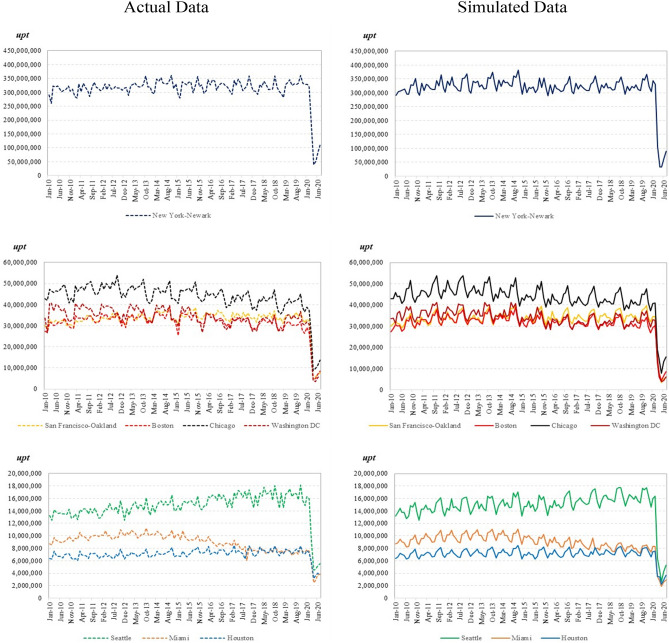
Simulation results for select UZAs. The figures on the left shows the actual UPT (unlinked passenger trips) in dashed lines, and the figures on the right shows the simulated UPT using the model in solid lines. UZAs simulated include New York-Newark, San Francisco-Oakland, Boston, Chicago, Washington DC, Seattle, Miami, and Houston. The UZAs are grouped in three sets by the level of UPT.

Figure 7 depicts the actual data alongside the fitted results of the AB estimation for several select UZAs. The figure panels are sorted by scale to better indicate impact, largely because some regions have far more public transit use than others; i.e., New York-Newark is, by far, the largest UZA nationwide in terms of unlinked passenger trips.

## Conclusions: extensions

We investigated the connection between the choice of transportation mode and the probability of COVID-19 transmission. This interplay will likely influence the choice of transportation means for years to come. The data highlights important connections between population density, public transportation use, and likelihood of transmission. Arellano-Bond and Instrumental Variables estimation techniques confirm a strong correlation between COVID-19 incidence and use of public transport use. We found strong evidence that aggregate public transit ridership in each UZA we studied has been negatively impacted by COVID-19 and by the policy responses to the pandemic. At the same time, ridership was positively impacted by higher employment levels and a rising price of gasoline. These findings highlight that concerns about disease transmission and COVID-19-related policies have had a negative effect on ridership which is over and above the adverse effect from the observed reduction in employment. Our analysis could be extended in several ways. As we concentrated on urban areas, we did not consider additional challenges affecting commuters in the countryside, where alternative means of transport might be rare, or non-existent. Similarly, in deriving the value of avoiding transmission (VAT), we could consider several kinds of heterogeneity among commuters. Such heterogeneity might lead to different thresholds regarding attitudes towards transmission risk. In future research, we could address explicitly how such decisions are made within different income groups, with some low-income commuters having few alternative choices.

A natural question to ask is how long it will take after COVID-19 incidents subside before demand for public transportation returns to normal levels. Given that fear and psychological factors are at play, and given evidence that repeated pandemics are becoming more frequent in recent decades, the “shock” might have a permanent component. This, in turn, would have implications for fuel use, congestion, accident frequency, and air quality. More vulnerable communities might be disproportionally affected as a result. Policy planning is needed to design our post-COVID-19 transportation future.

## Supplementary Information

Supplementary Information.
