# Supplementary material for: COVID-19 and the value of safe transport in the United States
Source: Sci Rep. 2021 Nov 4;11:21707. doi: 10.1038/s41598-021-01202-9 (PMC8569113; doi:10.1038/s41598-021-01202-9)
Supplement: Supplementary file 1 — Supplementary Information. [file 41598_2021_1202_MOESM1_ESM.pdf]

COVID-19 and the Value of Safe Transport in the United States

by

Kenneth B Medlock, III, Ted Temzelides, and Shih Yu (Elsie) Hung*

Center for Energy Studies

James A. Baker III Institute for Public Policy

Rice University

October 10, 2021

Keywords: Public transport, commuting, COVID-19, Future of work, energy use

Appendix

Figure A1. COVID-19 cases and the public transit system in Bay area, 6/30/20. The heat map suggests COVID-19 cases by zip code (in shades of orange) overlaid with public transit routes. The number of cases is the lowest in pale orange and highest in dark red The map was created by the authors with QGIS 3.12 (https://qgis.org/en/site/forusers/download.html)

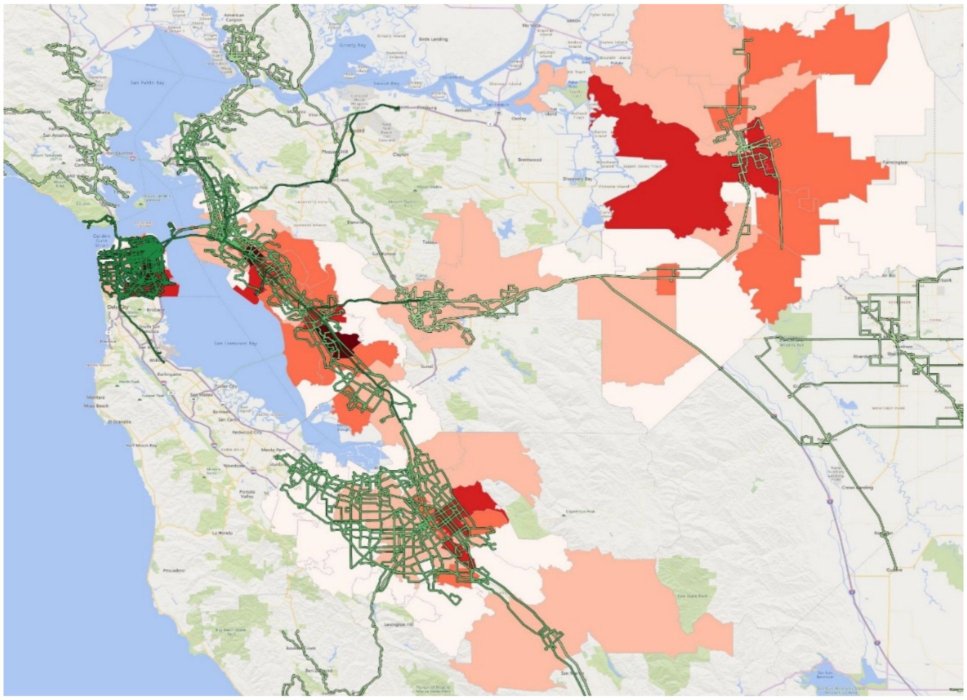

Figure A2. COVID-19 cases and the public transit system in Seattle, 6/30/20. The heat map suggests COVID-19 cases by zip code (in shades of orange) overlaid with public transit routes.

The number of cases is the lowest in pale orange and highest in dark red The map was created by the authors with QGIS 3.12 (https://qgis.org/en/site/forusers/download.html)

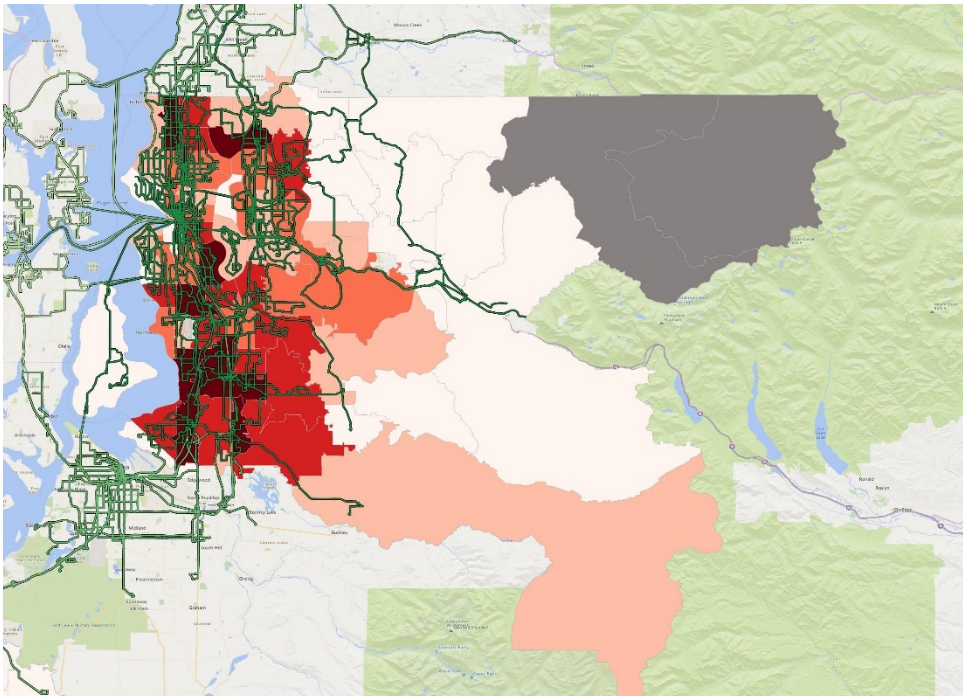

Figure A3. COVID-19 cases and the public transit system in San Diego, 6/30/20. The heat map suggests COVID-19 cases by zip code (in shades of orange) overlaid with public transit routes. The number of cases is the lowest in pale orange and highest in dark red The map was created by the authors with QGIS 3.12 (https://qgis.org/en/site/forusers/download.html)

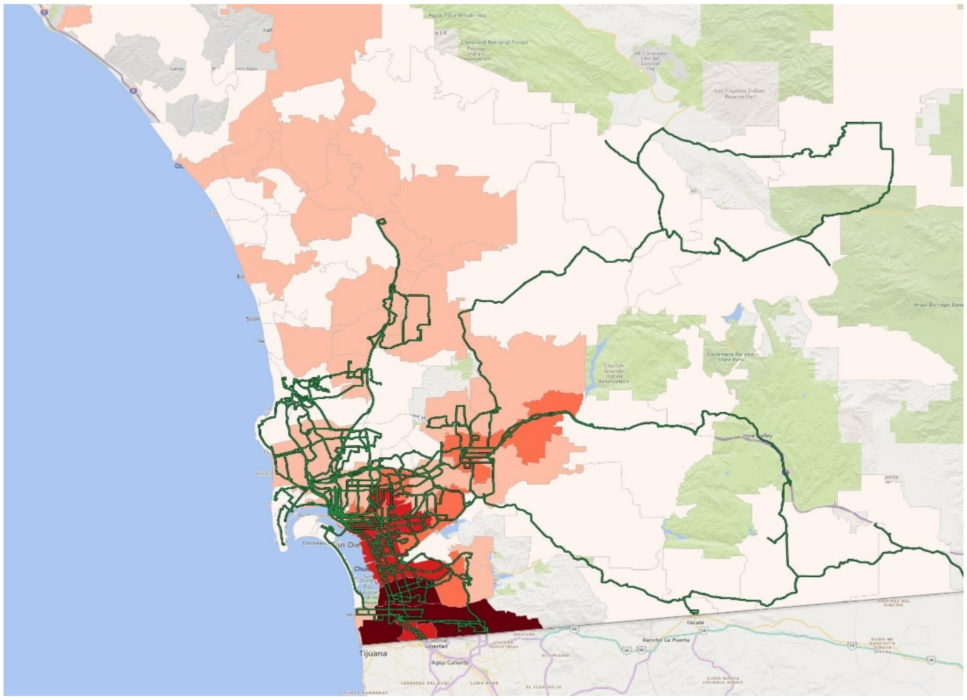

Figure A4. COVID-19 cases and the public transit system in St. Louis, 6/30/20. The heat map suggests COVID-19 cases by zip code (in shades of orange) overlaid with public transit routes. The number of cases is the lowest in pale orange and highest in dark red The map was created by the authors with QGIS 3.12 (https://qgis.org/en/site/forusers/download.html)

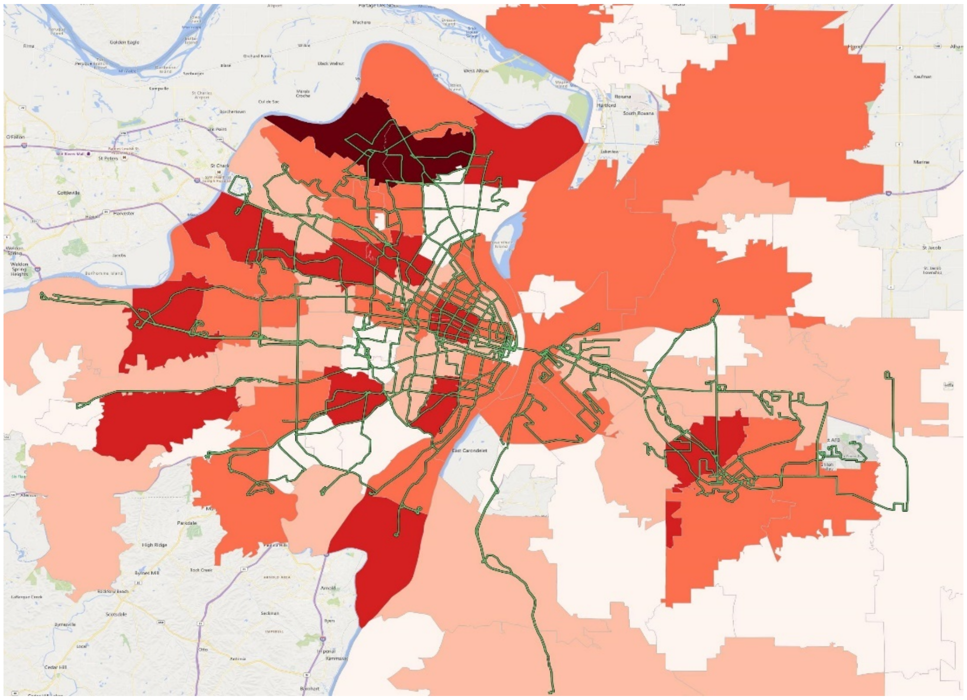

Table A1. Estimation Results

	Parameter	IV<br>Parameter Estimate<br>*standard error*	AB<br>Parameter Estimate<br>*standard error*
Constant	α_0<br>*std err*	4.3533<br>*0.1760*	4.2303<br>*0.1973*
ln $UPT_{i,t-1}$	α_1<br>*std err*	0.6370<br>*0.0075*	0.6173<br>*0.0080*
$DevPctHm_{i,t}$	α_2<br>*std err*	-1.4246<br>*0.0303*	-1.5767<br>*0.0329*
$NewCOVID_{i,t}$	α_3<br>*std err*	-0.0003<br>*0.0001*	-0.0013<br>*0.0002*
ln $N_{i,t}$	α_4<br>*std err*	0.0488<br>*0.0256*	0.1140<br>*0.0293*
ln $P_{gas,i,t}$	α_5<br>*std err*	0.1244<br>*0.0089*	0.1276<br>*0.0096*
Jan	α_6<br>*std err*	0.1248<br>*0.0069*	0.1230<br>*0.0066*
Feb	α_7<br>*std err*	0.0923<br>*0.0069*	0.0937<br>*0.0064*
Mar	α_8<br>*std err*	0.1583<br>*0.0070*	0.1668<br>*0.0065*
Apr	α_9<br>*std err*	0.0327<br>*0.0068*	0.0390<br>*0.0065*
May	α_{10}<br>*std err*	0.0501<br>*0.0068*	0.0544<br>*0.0065*
Jun	α_{11}<br>*std err*	-0.0092<br>*0.0068*	-0.0085<br>*0.0065*
Jul	α_{12}<br>*std err*	0.0175<br>*0.0069*	0.0177<br>*0.0066*
Aug	α_{13}<br>*std err*	0.1449<br>*0.0070*	0.1423<br>*0.0067*
Sep	α_{14}<br>*std err*	0.1313<br>*0.0069*	0.1308<br>*0.0066*
Oct	α_{15}<br>*std err*	0.1842<br>*0.0069*	0.1848<br>*0.0066*
Nov	α_{16}<br>*std err*	0.0086<br>*0.0069*	0.0109<br>*0.0066*
Wald $\chi^2(16)$		9.33E+07	27330.55
R^2		0.9893	
# Observations		11656	11656
# Groups		94	94
